# A silver lining for 24-hydroxycholesterol in Alzheimer's disease: The involvement of the neuroprotective enzyme sirtuin 1

**DOI:** 10.1016/j.redox.2018.05.009

**Published:** 2018-05-22

**Authors:** Gabriella Testa, Erica Staurenghi, Serena Giannelli, Simona Gargiulo, Michela Guglielmotto, Massimo Tabaton, Elena Tamagno, Paola Gamba, Gabriella Leonarduzzi

**Affiliations:** a Department of Clinical and Biological Sciences, University of Turin, Orbassano, Turin, Italy; b Neuroscience Institute of Cavalieri Ottolenghi Foundation (NICO), University of Turin, Orbassano, Turin, Italy; c Department of Neuroscience, University of Turin, Turin, Italy; d Unit of Geriatric Medicine, Department of Internal Medicine and Medical Specialties (DIMI), University of Genoa, Genoa, Italy

**Keywords:** 24-OH, 24-hydroxycholesterol, 27-OH, 27-hydroxycholesterol, 7-K, 7-ketocholesterol, AD, Alzheimer's disease, APP, amyloid precursor protein, Aβ, amyloid beta, DHE, dihydroethidium, ICV, intracerebroventricular, NFT, neurofibrillary tangles, ROS, reactive oxygen species, siRNA, small interfering RNA, SIRT1, sirtuin 1, 24-hydroxycholesterol, Sirtuin 1, Tau, Oxysterols, Alzheimer's disease

## Abstract

It is now established that cholesterol oxidation products (oxysterols) are involved in several events underlying Alzheimer's disease (AD) pathogenesis. Of note, certain oxysterols cause neuron dysfunction and degeneration but, recently, some of them have been shown also to have neuroprotective effects. The present study, which aimed to understand the potential effects of 24-hydroxycholesterol (24-OH) against the intraneuronal accumulation of hyperphosphorylated tau protein, stressed these latter effects. A beneficial effect of 24-OH was demonstrated in SK-N-BE neuroblastoma cells, and is due to its ability to modulate the deacetylase sirtuin 1 (SIRT1), which contributes to preventing the neurotoxic accumulation of the hyperphosphorylated tau protein. Unlike 24-OH, 7-ketocholesterol (7-K) did not modulate the SIRT1-dependent neuroprotective pathway. To confirm the neuroprotective role of 24-OH, in vivo experiments were run on mice that express human tau without spontaneously developing tau pathology (hTau mice), by means of the intracerebroventricular injection of 24-OH. 24-OH, unlike 7-K, was found to completely prevent the hyperphosphorylation of tau induced by amyloid β monomers. These data highlight the importance of preventing the loss of 24-OH in the brain, and of maintaining high levels of the enzyme SIRT1, in order to counteract neurodegeneration.

**Graphical abstract:**

A hypothetical scheme of the molecular mechanisms underlying the effects of 24-OH on hyperphosphorylated tau accumulation.

**Figure:**
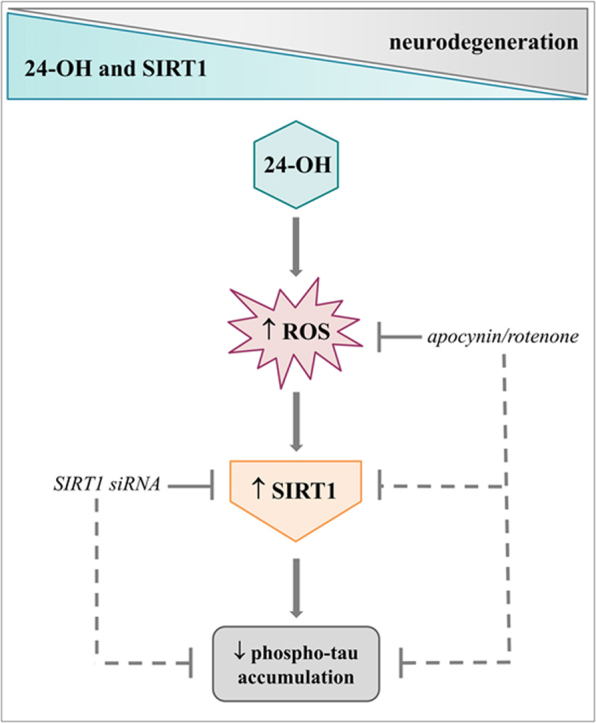

## Highlights

• 24-OH, unlike 7-K, upregulates the neuroprotective enzyme SIRT1.
• 24-OH induces a cell redox imbalance leading to SIRT1 activation.
• 24-OH prevents the accumulation of hyperphosphorylated tau.

## 1 Introduction

Alzheimer's disease (AD) is one of the biggest health and social care challenges world-wide. The difficulty of early diagnosis of AD, and the lack of effective therapies, provide incentives for the development of new strategies to prevent or slow this disabling disease.

In AD, hyperphosphorylated tau and Aβ accumulate, respectively generating intraneuronal neurofibrillary tangles (NFT) and extracellular senile plaques. Regarding the formation of these two lesions, findings concerning which comes first are discordant; however, growing evidence recognizes the very early onset of tau pathology [1], [2].

In normal conditions, tau is involved in microtubule assembly and stability, and in neuronal survival; under pathological conditions, tau undergoes excessive phosphorylation and acetylation, resulting in aggregation in toxic insoluble oligomers that destabilize the microtubules. In particular, the hyperphosphorylation of tau reduces its binding to the microtubules, whereas tau hyperacetylation inhibits ubiquitin-mediated proteolysis of phosphorylated tau, thereby promoting its self-aggregation. Consequently, microtubules depolymerize and NFT accumulate inside neurons leading to neurodegeneration [3].

In the last few years, a close link between brain cholesterol dyshomeostasis and AD has been recognized, but it remains unclear at what stage the cholesterol biosynthetic pathway is perturbed, and how this contributes to AD pathogenesis. Thanks to a large body of research, it is now believed that one of the main triggers in AD is oxidized cholesterol in the form of oxysterols [4], [5].

Cholesterol homeostasis in the normal brain is tightly regulated: cholesterol is synthetized *de novo* by astrocytes and then loaded with apolipoprotein E which drives it to neurons. Once internalized by neurons, excess cholesterol is oxidized into 24-hydroxycholesterol (24-OH) by the enzyme CYP46A1 or into 27-hydroxycholesterol (27-OH) by the enzyme CYP27A1. Of note, 24-OH is one of the main oxysterols most closely involved in AD pathogenesis since its levels are markedly decreased, because of the loss of neurons expressing CYP46A1 [6].

Indeed, previous research by the present authors on the frontal and occipital cortex of AD patients, classified by the Braak staging system of neurofibrillary pathology, have clarified the association between the concentrations of oxysterols in the brain and progression of the disease [7]. These data underline the significant reduction of 24-OH levels, unlike the other oxysterols, in the brain during the progression of AD, and it is likely that its loss is a factor in accelerating development of the disease. Of note, it was observed that expression levels of the gene coding for the enzyme responsible for the formation of 24-OH, CYP46A1, decreased in parallel with its loss. Conversely, other oxysterols significantly increased, including 27-OH, an oxysterol of enzymatic origin like 24-OH, and some oxysterols deriving from cholesterol autooxidation, such as 7-ketocholesterol (7-K), 25-hydroxycholesterol, 7α- and 7β-hydroxycholesterol, α- and β-epoxycholesterol, 4α- and 4β-hydroxycholesterol [7]. These observations are in agreement with other studies that correlate specific oxysterols with disease state [8], [9].

The idea is now emerging that certain oxysterols, accumulating in the brain during AD, can act as friends and/or foes: it has clearly been observed that they cause neuron dysfunction and degeneration, contributing to neuroinflammation and amyloidogenesis, but, conversely, some oxysterols can also be neuroprotective. The underlying molecular mechanisms, however, remain to be fully elucidated, in particular concerning the beneficial effects.

AD is characterized by both amyloid and tau pathology [10]. The link between altered cholesterol metabolism and amyloid beta (Aβ) accumulation has been extensively investigated [11], [12], [13], [14], but its relationship with tau pathology is currently almost entirely unknown, with very few exceptions [15], [16], [17].

This paper aimed to compare the effects of 24-OH and 7-K, on tau phosphorylation, a field hitherto completely unexplored.

Intraneuronal accumulation of NFT made of hyperphosphorylated tau protein is directly correlated with cognitive decline in AD and other primary tauopathies, making it crucial to find neuroprotective pathways that might prevent or reduce the hyperphosphorylation of tau. In particular, the focus here was on the sirtuin 1 (SIRT1)-dependent neuroprotective pathway, because it appears to display protective effects against AD progression, and it might be closely involved in the neurofibrillary pathology. The deacetylase SIRT1 is considered pro-survival, because of its several beneficial effects, including the ability to inhibit NFT and Aβ plaque accumulation [18].

Interestingly, the present group recently demonstrated that expression of SIRT1 in the brain drastically decreases with the progression of AD, suggesting that, in parallel with the loss of 24-OH, its reduction might play a key role in AD pathology. Moreover, it was observed that, in the AD-affected brain, the accumulation of hyperphosphorylated tau co-occurs with the reduction of SIRT1 activity. It also appears clear that SIRT1 reduction correlates with neuroinflammation in AD. In particular, elevated inflammatory molecule levels have been detected in the brain of AD patients in the earlier phases of AD, precisely when SIRT1 levels begin to shrink [7], [19], [20].

To investigate whether the reduction of 24-OH in the AD brain might be responsible for the concurrent reduction of SIRT1 and the consequent tau hyperphosphorylation, the ability of 24-OH to modulate the SIRT1-dependent neuroprotective pathway was analyzed in SK-N-BE neuroblastoma cells. It was shown that 24-OH, unlike 7-K, activates the SIRT1-dependent neuroprotective pathway, by inducing the intracellular generation of reactive oxygen species (ROS). Consequently, 24-OH favors tau deacetylation and prevents intracellular accumulation of hyperphosphorylated tau.

It is worthy of note that the strong neuroprotective action of 24-OH was also confirmed, in vivo, in mice expressing human tau (hTau mice) that produce tau aggregates after Aβ monomer administration. The hTau mice were subjected to an intracerebroventricular (ICV) injection of 24-OH which has been shown to be effective against tau hyperphosphorylation induced by Aβ.

For the first time, the results demonstrate an important role for 24-OH in reducing hyperphosphorylated tau accumulation, a sort of “silver lining” for this oxysterol, which thus far has been observed to exert mainly negative effects.

## 2 Materials and methods

### 2.1 Cell culture and treatments

SK-N-BE neuroblastoma cells were maintained in RPMI 1640 medium supplemented with 2 mM glutamine, 10% FBS, 1% non-essential aminoacids and 1% antibiotic mixture at 37 °C with 5% CO_2_. Cells were treated with 1 μM 24-OH (Avanti Polar Lipids Inc., Alabaster, AL, USA) or with 1 µM 7-K (Steraloids, Newport, RI, USA), both dissolved in ethanol. In some experiments, cells were pretreated with 300 μM apocynin 1 h before oxysterol treatment, or were cotreated with 2.5 μM rotenone.

### 2.2 Mice and ICV injections

Mice expressing human tau, hTau mice (Mapt ^tm1(EGFP)Klt^Tg(MAPT) 8cPdav/J; #004808, The Jackson Laboratory, Bar Harbor, ME, USA), were crossed with tau knock-out (KO) mice (Mapt ^tm1(EGFP)Klt^/J; #004779, Jackson Laboratory), to obtain pregnant females carrying hTau fetuses [21]. The mice were genotyped by PCR assay as described by Manassero and colleagues [22]. They were maintained on a Swiss Webster/129/SvJae/C57BL/6 background [21], and kept on a 12 h light/dark cycle with food and water available ad libitum.

All experimental procedures on live animals were performed under the supervision of a licensed veterinarian, in agreement with: i) European Communities Council Directive (November 24, 1986; 86/609/EEC); ii) Italian Ministry of Health and University of Turin's institutional guidelines on animal welfare (DL 116/92 on Care and Protection of living animals undergoing experimental or other scientific procedures; authorization No. 17/2010-B, June 30, 2010); iii) the ad hoc Ethical Committee of the University of Turin (http://www.unito.it/unitoWAR/page/istituzionale/ricerca1/Ricerca_comitato1).

For the experimental procedures, 2 month-old male hTau mice were used; under isoflurane O_2_/N_2_O anesthesia, the mice (n = 30) were ICV injected with 1 μM 24-OH or 1 μM 7-K for 4 days, and then with 0.2 μM Aβ_1–42_ monomers (AnaSpec, Seraing, Belgium) or saline (control mice) for 3 h. The lyophilized synthetic Aβ peptides were dissolved in 1% NH_4_OH to get a clear solution and stored at −20 °C in aliquots. Monomeric preparations were brought to 0.2 μM (final concentration) with sterile double distilled water, centrifuged at 10,000 g for 10 min to remove possible aggregates. The quality of Aβ preparations was routinely controlled using atomic force microscopy [22]. Both 24-OH and 7-K were dissolved in ethanol at the final concentration of 1 μM for ICV injection. Coordinates used for injection were: anteroposterior, −0.5 mm; lateral, 1.2 mm relative to bregma and dorsoventral, 1.7 mm from the dural surface. The method was validated by injecting one mouse with Trypan blue (1 µL).

### 2.3 Small interfering RNA (siRNA) transfection

SIRT1 siRNA was used for transient gene knockdown study (Life Technologies, Thermo Fisher Scientific, Monza, Italy). Transfection of SIRT1 siRNA was performed following the manufacturer's instructions. Briefly, 25 µL of 100 nM siRNA were mixed with 25 µL of transfection reagent solution containing 1.5 µL of trasfection reagent (Lipofectamine^®^ RNAiMAX Reagent, Life Technologies) and left for 10 min in RPMI medium with 1% FBS and without antibiotics. After 48 h of reverse transfection, the cells (6 × 10^4^/500 µL) were incubated with 1 μM 24-OH for 15 h. Total RNA was isolated from the cells and used for quantitative RT-PCR as described below. The transfection efficiency, validated by quantitative RT-PCR, was approximately 77%. Total protein extract was obtained from the cells following the protocol described below.

### 2.4 RNA extraction and cDNA synthesis

Total RNA was extracted using TRIzol Reagent (Life Technologies) following the manufacturer's instructions. RNA was dissolved in RNase-free water fortified with RNase inhibitors (RNaseSUPERase-In; Life Technologies). The amount and purity (A260/A280 ratio) of the extracted RNA were assessed spectrophotometrically. cDNA was synthesized by reverse transcription from 2 μg RNA with a commercial kit and random primers (High-Capacity cDNA Reverse Transcription Kit; Life Technologies) following the manufacturer's instructions.

### 2.5 Real time RT-PCR

Singleplex real-time RT-PCR was performed on 30 ng cDNA using TaqMan Gene Expression Assay kits prepared for human SIRT1 and β_2_-microglobulin, TaqMan Fast Universal PCR Master Mix, and 7500 Fast Real-Time PCR System (Life Technologies). The oligonucleotide sequences are not revealed by the manufacturer because of proprietary interests. The cycling parameters were as described by Gamba and colleagues [11]. The fractional cycle number (Ct) at which fluorescence passes the threshold in the amplification plot of fluorescence signal versus cycle number was determined for each gene considered. The results were then normalized to the expression of β_2_-microglobulin. Relative quantification of target gene expression was achieved with a mathematical method [23].

### 2.6 Protein extraction and Western blotting

Cells were lysed in ice-cold lysing buffer (1 mL PBS fortified with 10 µL Triton X-100, 10 µL SDS 10%, 5 µL DTT 1 M, 6 µL PMSF 0.1%, and 10 µL aprotinin) (30 min), sonicated on ice, and then centrifugated at 17,860 g (15 min). Fresh frozen brains were mechanically homogenized in ice-cold buffer (25 mM Tris-HCl pH 7.4, 150 mM NaCl, 1 mM EGTA, 1 mM EDTA, 1 mM PMSF, phosphatase and protease inhibitors) and then centrifuged at 10,000 g (15 min) to isolate soluble proteins.

To analyze the protein levels, 50 µg (cell lysates) or 20 µg (brain lysates) of total proteins were separated by electrophoresis in 8% denaturing SDS/polyacrylamide gel, then transferred to Amersham Hybond PVDF membrane (GE Healthcare Europe, Milan, Italy). After saturation of non-specific binding sites with 5% non-fat milk in Tris-buffered saline 1x-Tween 20 0.05% for 1 h, the membrane was immunoblotted overnight at 4 °C with the primary antibodies against SIRT1 (1:1000) (Cell Signaling Technology, Denver, CO, USA or Merk-Millipore, Darmstadt, Germany), tau (1:750), phospho-tau (1:400) (Santa Cruz Biotechnology Inc., Denver, CO, USA), and acetyl-tau (1:200) (AnaSpec), AT8 (1:500) (Innogenetics, Alpharetta, GA, USA) or tau5 (1:500) (Merk-Millipore). The membranes were stripped and re-immunoblotted with anti-α-tubulin (1:1000) (Cell Signaling Technology) or with anti-β-actin (1:5000) primary antibody (Sigma-Aldrich, St. Louis, MO, USA).

The immunoreactive bands were visualized through enhanced chemiluminescence using Clarity Western ECL Substrate (Bio-Rad Laboratories Inc.) following the manufacturer's protocol. The bands were quantified by densitometric analysis using Image J 1.43 software. The results were evaluated as relative units determined by normalization of the density of each band to that of the corresponding α-tubulin or β-actin protein band.

### 2.7 Measurement of intracellular ROS

The production of ROS, principally superoxide anion (O_2_^.-^), was detected by dihydroethidium (DHE) fluorescence staining (Sigma-Aldrich). After treatment, the cells were washed and resuspended with RPMI-1640 medium (fortified with 2% FBS) and incubated for 30 min in the dark with 5 μM DHE at 37 °C. Fluorescence was immediately detected with a confocal laser microscope (Zeiss LSM 510; Carl Zeiss S.p.A., Arese, Milan, Italy) setting the exciting laser band to 543 nm, and using a 560–615 nm band-pass emission filter (plan neofluar lens 40×/0.75). All images were processed using LSM 510 Image Examiner software (Carl Zeiss S.p.A.).

### 2.8 Analysis of SIRT1 and phospho-tau by immunofluorescence

Cells were grown on glass slides and, after treatments, specimens were fixed in 4% formalin for 15 min and then washed with 0.1 M PBS. After blocking nonspecific binding sites with 0.1 M PBS containing 5% goat serum, 3% BSA, and 0.3% Tween 20, for 30 min, slides were incubated in the presence of primary antibodies against SIRT1 (1:150) or against phospho-tau (1:150) (Santa Cruz Biotechnology Inc.) for 1.5 h, and then with specific secondary antibodies (1:300) conjugated with fluorescein isothiocyanate (FITC) fluorochrome (Alexa Fluor, Molecular Probes, Life Technologies) for 1 h. Slides, mounted with Fluoroshield (Sigma-Aldrich), were observed through a LSM 510 confocal laser microscope (Carl Zeiss S.p.A.) (488 nm exciting laser band and emission passing through a long-pass 505–550 filter, lens 40×/0.75). The images were processed using LSM 510 Image Examiner software (Carl Zeiss S.p.A.).

### 2.9 Statistical analysis

All values are expressed as means ± SD. Data were analyzed statistically using one-way ANOVA with Bonferroni's post test for multiple comparisons. Differences at P < 0.05 were considered statistically significant. Calculations were performed using GRAPHPAD INSTAT3 software (GraphPad Software Inc., San Diego, CA, USA).

## 3 Results

### 3.1 24-OH, unlike 7-K, upregulates both expression and synthesis of SIRT1

**Fig. 1:**
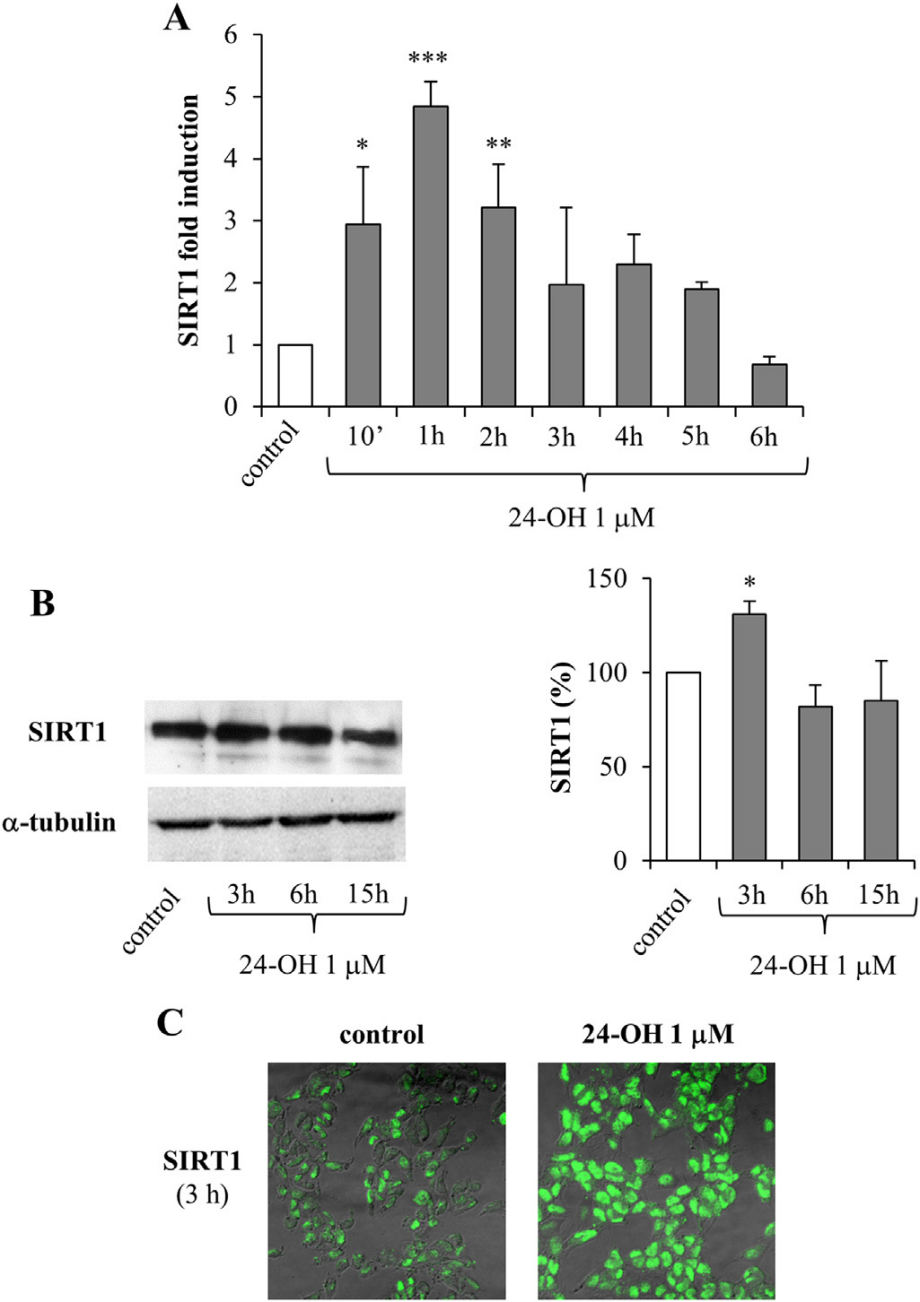
Effect of 24-OH on SIRT1 expression and synthesis in SK-N-BE cells. (A) Gene expression was quantified in cells treated with 24-OH. Data, normalized to β2-microglobulin, are expressed as mean values ± SD of 4 experiments. *P < 0.05, **P < 0.01, and ***P < 0.001 vs control. (B) SIRT1 protein levels were analyzed in cells treated with 24-OH. The blot shown is representative of 3 experiments. The histograms represent mean values ± SD of 3 experiments. SIRT1 densitometric measurements were normalized against the corresponding α-tubulin levels and expressed as percentage of control value. *P < 0.05 vs control. (C) After cell treatment with 24-OH, SIRT1 protein levels were detected by confocal laser microscopy. The images are representative of 3 experiments.

**Fig. 2:**
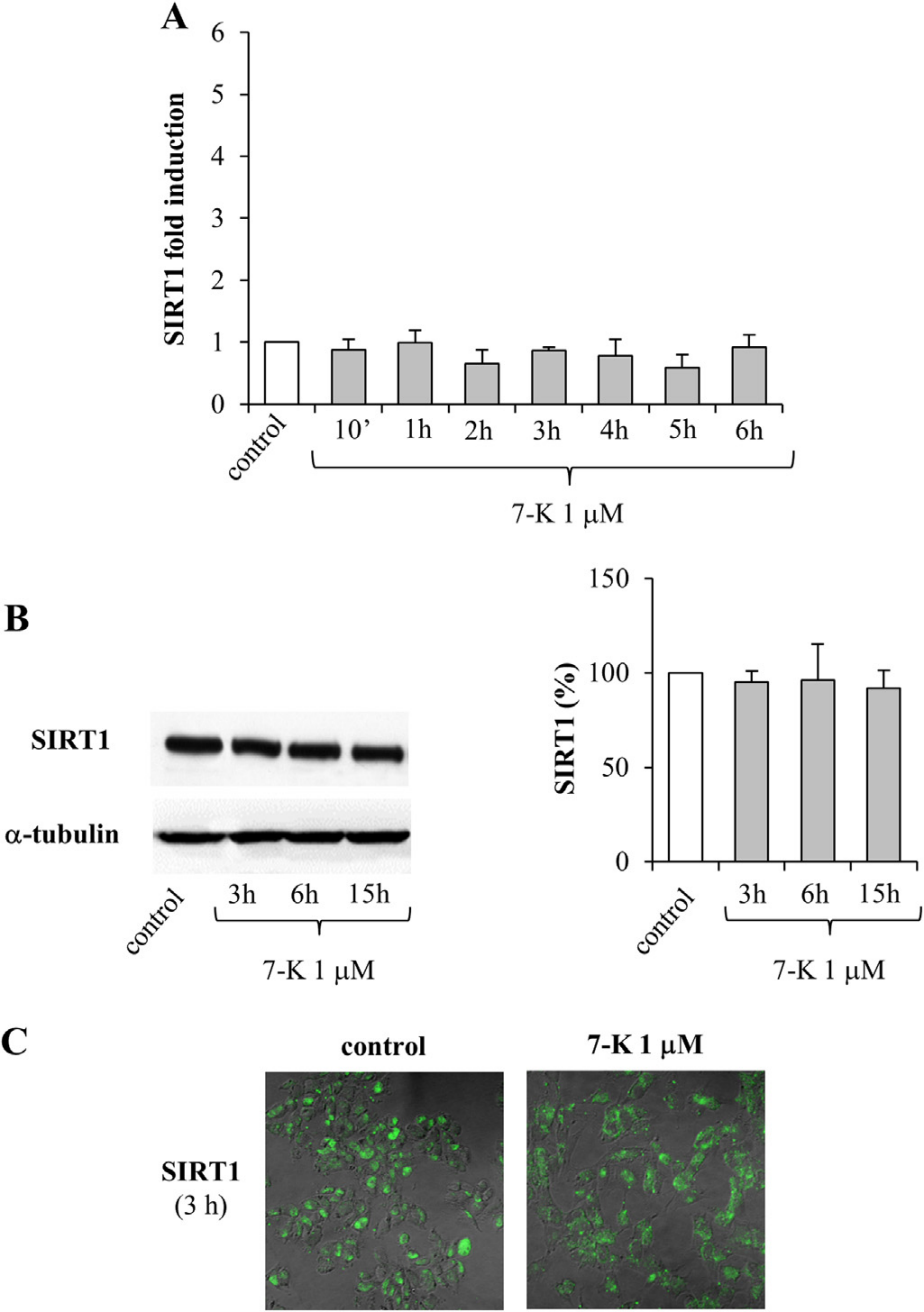
Effect of 7-K on SIRT1 expression and synthesis in SK-N-BE cells. (A) Gene expression was quantified in cells treated with 7-K. Data, normalized to β2-microglobulin, are expressed as mean values ± SD of 4 experiments. (B) SIRT1 protein levels were analyzed in cells treated with 7-K. The blot shown is representative of 3 experiments. The histograms represent mean values ± SD of 3 experiments. SIRT1 densitometric measurements were normalized against the corresponding α-tubulin levels and expressed as percentage of control value. (C) After cell treatment with 7-K, SIRT1 protein levels were detected by confocal laser microscopy. The images are representative of 3 experiments.

With the aim of determining whether the oxysterols 24-OH and 7-K are able to modulate the SIRT1-dependent neuroprotective pathway, the expression and synthesis of SIRT1 were measured after treatment of neuroblastoma SK-N-BE cells either with 24-OH (Fig. 1) or with 7-K (Fig. 2), both at the physiopathological concentration of 1 µM.

SIRT1 gene expression was found to be significantly upregulated only by 24-OH, the effect being evident after 10 min and continuing to 2 h, with a maximum at 1 h; after 2 h cell treatment with 24-OH there was a progressive reduction in SIRT1 expression levels, until control levels were reached (6 h) (Fig. 1A). Conversely, 7-K did not induce SIRT1 expression at any time (Fig. 2A).

As regards SIRT1 protein levels, it emerged that 24-OH induced its synthesis after 3 h cell treatment, as demonstrated by Western blotting (Fig. 1B) and immunocytochemistry (Fig. 1C). Again, 7-K did not modulate protein levels of SIRT1 in neurons (Fig. 2B and C).

### 3.2 24-OH, unlike 7-K, reduces tau acetylation, thus preventing hyperphosphorylated tau accumulation

To check whether modulation of the expression and synthesis of the deacetylase SIRT1 by 24-OH (as shown in Fig. 1) is actually followed by its activation, the levels of acetylated tau (acetyl-tau) were analyzed by Western blotting. Levels of total and phosphorylated tau (phospho-tau) were also measured, in order to verify the oxysterol's effect in modulating NFT formation.

**Fig. 3:**
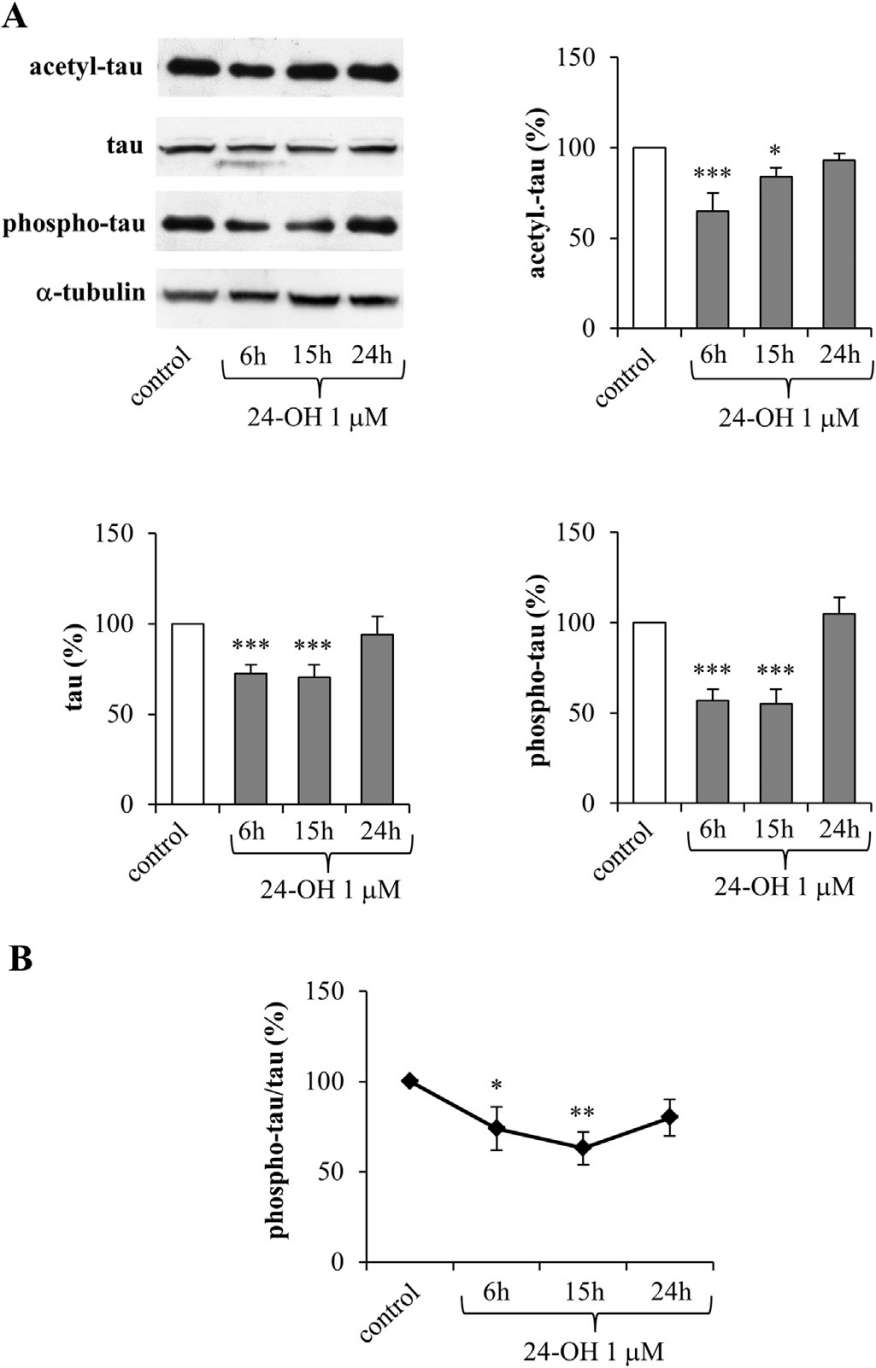
Effect of 24-OH on the levels of acetylated, phosphorylated, and total tau in SK-N-BE cells. (A) Acetyl-tau, phospho-tau, and total tau levels were analyzed in cells treated with 24-OH. Each blot shown is representative of 3 experiments. The histograms represent mean values ± SD of 3 experiments. Densitometric measurements were normalized against the corresponding α-tubulin levels and expressed as percentage of control value. *P < 0.05 and ***P < 0.001 vs control. (B) Phospho-tau/tau ratio derived from densitometric measurements. *P < 0.05 and **P < 0.01 vs control.

SK-N-BE cells were treated with 1 µM 24-OH for times ranging from 6 h to 24 h. 24-OH significantly reduced levels of both tau acetylation and tau phosphorylation (Fig. 3A). As regards acetyl-tau, it was significantly reduced after 6 and 15 h, and the consequent tau phosphorylation levels were also markedly inhibited after 6 and 15 h. Both acetyl- and phospho-tau were measured by normalizing their levels to α-tubulin content. Interestingly, 24-OH was also found to reduce tau protein levels after 6 and 15 h (Fig. 3A). This reduction is presumably due to tau deacetylation, which is known to favor tau proteasomal degradation [3]; this hypothesis is clearly supported by the evidence of the progressive reduction until 15 h of phospho-tau/tau ratio (Fig. 3B).

**Fig. 4:**
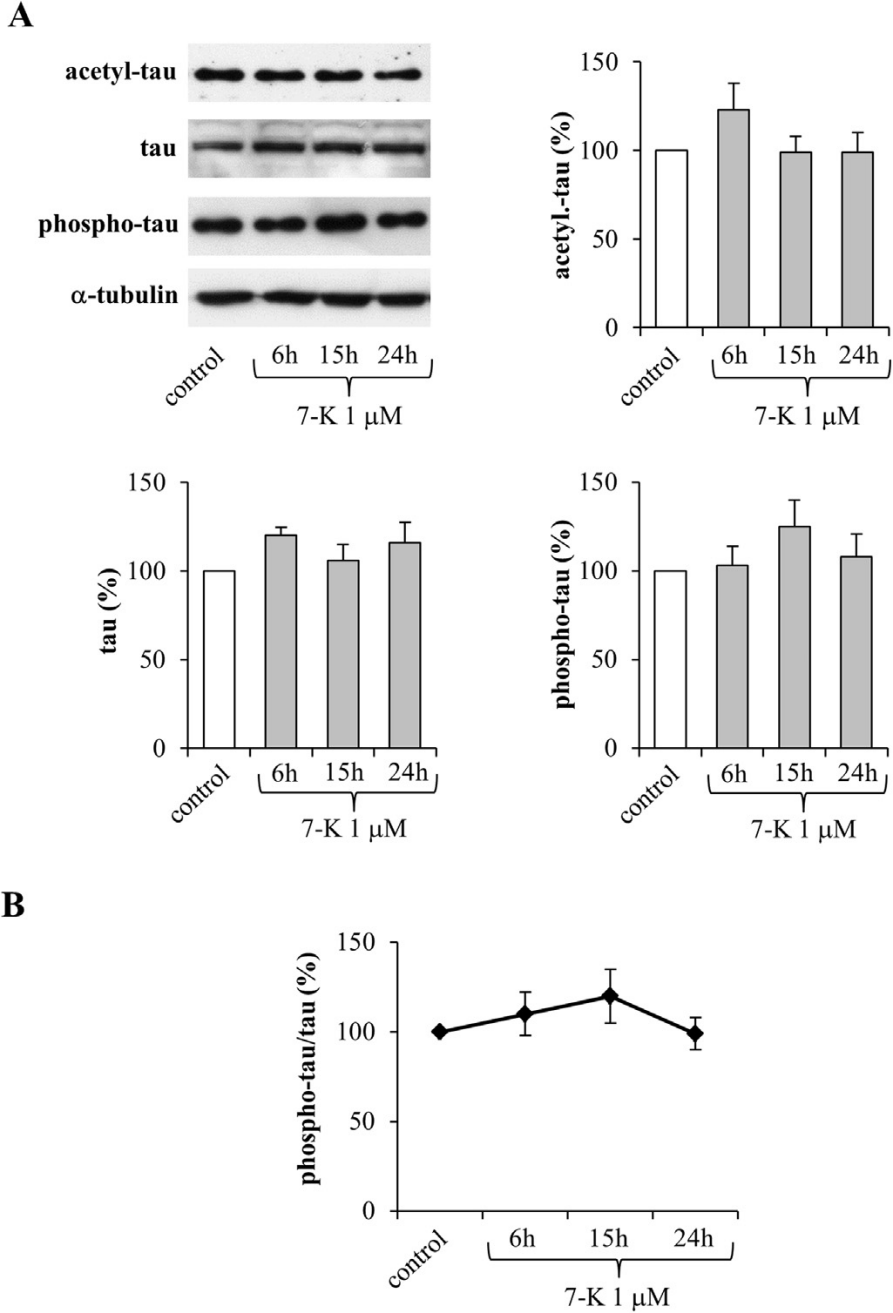
Effect of 7-K on the levels of acetylated, phosphorylated, and total tau in SK-N-BE cells. (A) Acetyl-tau, phospho-tau, and total tau levels were analyzed in cells treated with 7-K. Each blot shown is representative of 3 experiments. The histograms represent mean values ± SD of 3 experiments. Densitometric measurements were normalized against the corresponding α-tubulin levels and expressed as percentage of control value. (B) Phospho-tau/tau ratio derived from densitometric measurements.

The same experiments were carried out on cells treated with 1 µM 7-K (Fig. 4). Of note, 7-K affected neither tau acetylation, nor tau phosphorylation, nor total tau, confirming that this oxysterol is not able to activate the SIRT1-dependent pathway (Fig. 4A). Interestingly, it even appears to have an opposing effect: it causes a slight increase, evident although not significant, of both acetyl- and phospho-tau levels. Unlike 24-OH cell treatments, 7-K did not affect the ratio between phospho-tau and total tau (Fig. 4B).

### 3.3 24-OH increases ROS steady-state levels in SK-N-BE cells

**Fig. 5:**
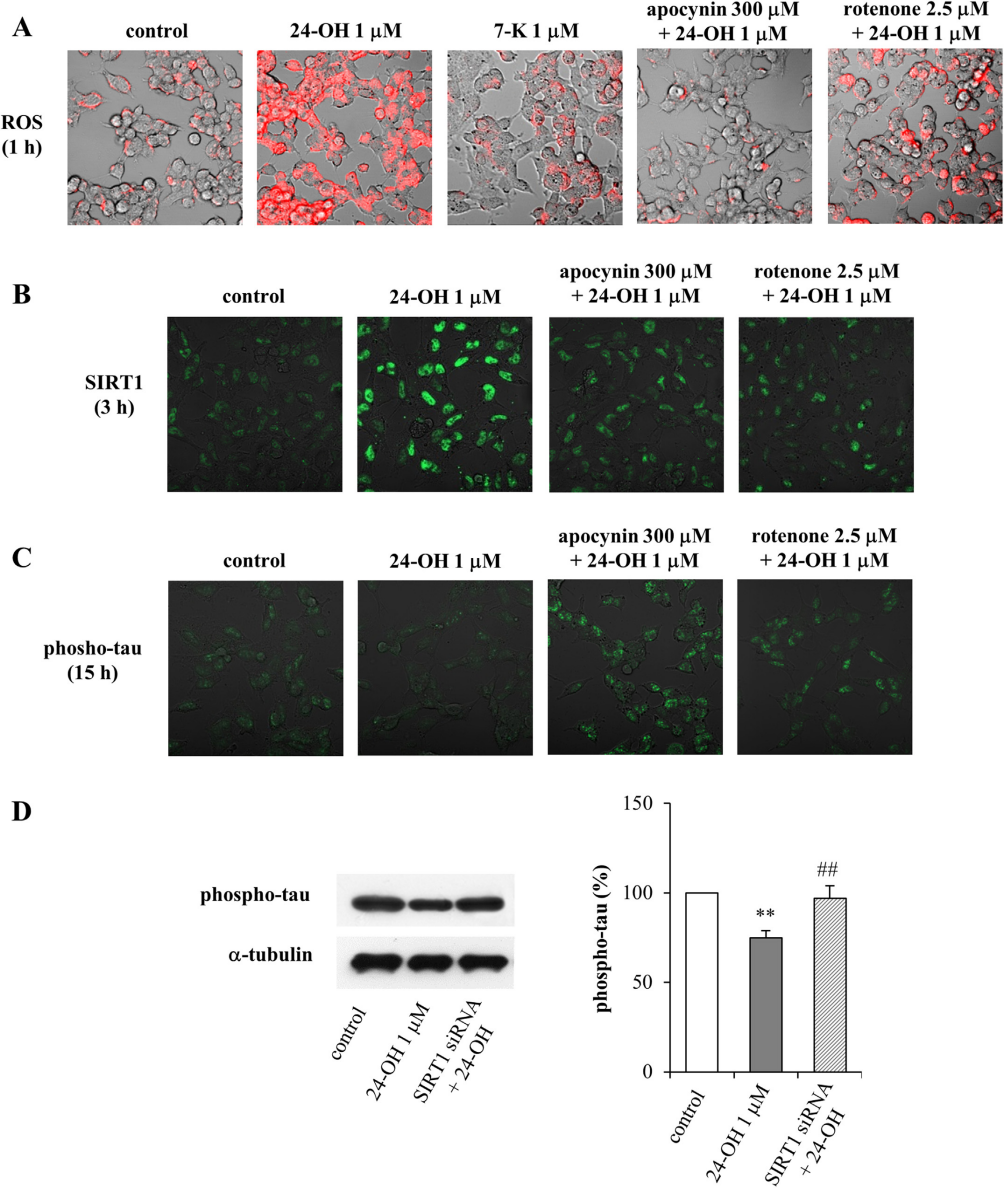
Effect of 24-OH and 7-K on ROS production and modulation of SIRT1 and phospho-tau levels by 24-OH in SK-N-BE cells. (A) Cells were treated with 24-OH or 7-K. The intracellular generation of ROS was monitored by DHE fluorescence staining. (B) The levels of SIRT1 and (C) phospho-tau were observed in cells incubated with 24-OH. Some cells were incubated with apocynin or with rotenone, and then treated with 24-OH. ROS, SIRT1, and phospho-tau levels were detected by confocal laser microscopy. The images are representative of 3 experiments. (D) Phospho-tau levels were analyzed in cells transfected with a specific SIRT1 siRNA and then incubated with 24-OH for 15 h. The blot is representative of 3 experiments and the histograms represent mean values ± SD of 3 experiments. Phospho-tau densitometric measurements were normalized against the corresponding α-tubulin levels and expressed as percentage of control value. ** p < 0.01 vs control; ## p < 0.01 vs 24-OH.

The incubation of SK-N-BE cells with 1 µM 24-OH induced, already after 1 h, a marked increase of ROS levels, as observed in DHE-stained cells. Conversely, incubation of the cells with 1 µM 7-K caused no ROS increase over control levels (Fig. 5A). Some evidence points to the ability of oxysterols to upregulate NADPH oxidase and to impair mitochondrial membrane potential, thus producing high levels of intracellular ROS [24], [25], [26]. On this basis, the involvement of NADPH oxidase and mitochondrial membrane potential derangement in the observed ROS production was verified. Neurons were treated with 24-OH for 1 h, in the presence or absence of 300 μM apocynin or 2.5 μM rotenone, specific inhibitors of NADPH oxidase and mitochondrial electron transport chain, respectively. Of note, apocynin and rotenone prevented ROS production (Fig. 5A).

In agreement with these data, NADPH oxidase activation and mitochondrial derangement were shown to be sources of ROS in 24-OH-treated cells (data not shown).

### 3.4 24-OH prevents hyperphosphorylated tau accumulation through the ROS/SIRT1-dependent neuroprotective pathway

The involvement of ROS production in SIRT1-dependent inhibition of phospho-tau intraneuronal accumulation was confirmed. Cells were pretreated (1 h) with apocynin (300 µM), or cotreated with rotenone (2.5 µM), and then with 24-OH (1 µM) for 3 h (Fig. 5B) or for 15 h (Fig. 5C). The increase of SIRT1 levels in neuroblastoma cells incubated with 24-OH was further confirmed by immunocytochemistry after 3 h cell treatment; this effect was completely prevented by the administration of the ROS inhibitors, either of apocynin or rotenone (Fig. 5B). As a consequence of SIRT1 positive modulation, the inhibition of tau phosphorylation was again demonstrated after 15 h cell treatment, and, also in this case, ROS production was shown to be a key step, since both inhibitors (apocynin and rotenone) counteracted the protective effect of 24-OH (Fig. 5C). In particular, in both experiments it was noted that 24-OH acts by inducing the activation of NADPH oxidase and mitochondrial derangement, thus leading to the release of ROS, which act as signal modulators, inducing the upregulation of the SIRT1-dependent pathway.

It was further demonstrated that the 24-OH-dependent neuroprotective effect, represented by the inhibition of tau hyperphosphorylation, actually depends on SIRT1 activation; when SIRT1 is silenced, tau phosphorylation is no longer prevented (Fig. 5D).

### 3.5 In vivo administration of 24-OH, but not of 7-K, prevents hyperphosphorylated tau accumulation in hTau mice by SIRT1 upregulation

**Fig. 6:**
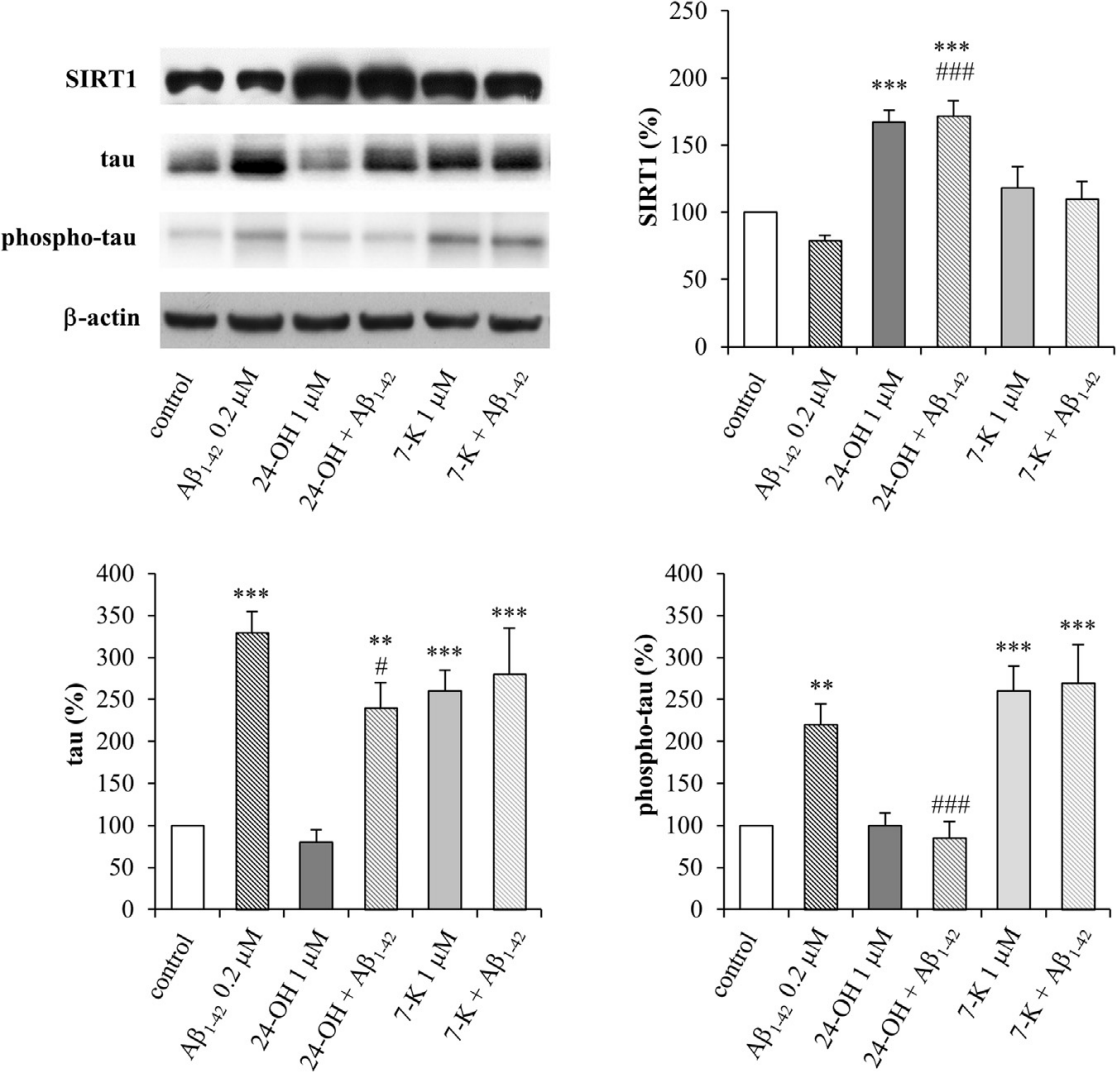
24-OH protects against phosphorylated and total tau accumulation induced by Aβ_1–42_ in mouse brain. hTau mice were ICV injected with 24-OH or 7-K for 4 days, and then with Aβ_1–42_ or saline (control) for 3 h. Representative blots of brain extracts, using SIRT1, AT8 or Tau5. Densitometric measurements were normalized against the corresponding β-actin levels and expressed mean values ± SD, n = 5 each group. **p < 0.01 and ***p < 0.001 vs control; #p < 0.05 and ### p < 0.001 vs Aβ_1–42_.

To determine whether 24-OH or 7-K induce tau phosphorylation in hTau mice, the levels of tau phosphorylation were measured using the AT8 antibody (Fig. 6). hTau mice injected ICV with monomeric preparations of Aβ_1–42_, which has been found to significantly increase tau phosphorylation [22], were used as positive controls. As expected, 3 h after the Aβ_1–42_ monomer injection, the hyperphosphorylation of tau significantly increased. Pretreatment with 24-OH (4 days) completely prevented the hyperphosphorylation of tau, whereas 7-K alone or in combination with Aβ_1–42_ mediated a significant increase of tau phosphorylation. The total tau levels were also evaluated (Fig. 6), and it emerged that Aβ_1–42_ significantly increased the total levels of tau. Pretreatment with 24-OH significantly but not completely decreased total tau levels, while 7-K alone or in combination with Aβ_1–42_ significantly increased them. The effect of 24-OH appears to be due to its capacity to increase SIRT1 levels in the brain of hTau mice, compared to hTau mice injected with Aβ_1–42_ or with 7-K (Fig. 6).

## 4 Discussion

Despite the many studies on the pathogenesis of AD, the exact sequence of events leading to neurodegeneration in the brain has not yet been fully clarified; however, in recent years increasing experimental evidence points to disordered cholesterol metabolism in the brain as one of the main drivers of AD development [4], [5].

Many studies have focused on understanding the molecular mechanisms whereby cholesterol oxidation contributes to AD development, and it has emerged that the behavior of oxysterols is without doubt peculiar: they are double-edged compounds, showing both detrimental and beneficial properties at the same time. In this connection, it has been demonstrated that the oxysterols are capable of triggering both death and survival signals within cells, depending on the experimental model [26], [27], [28], [29], [30].

The role of 24-OH in AD in some contexts is controversial and still debated. The effects exerted by 24-OH appear to depend on its concentration: high concentrations (25–50 µM) are toxic to neuroblastoma SH-SY5Y cells [31] while conversely pretreatment with low sub-lethal concentrations of 24-OH (1–10 µM), within the range observed in human brain, induces an adaptive response via activation of liver x receptor, and counteracts the 7-K-dependent cytotoxicity [32]. In another experimental model, it has been shown that 24-OH (1 µM) is not neurotoxic per se, while it markedly potentiates both the apoptotic and the necrogenic effects exerted by Aβ_1–42_ peptide on different neuronal cells. This effect was due to 24-OH's ability to enhance the binding of Aβ to neurons, and thus its intracellular accumulation, by amplifying the availability of a multireceptor complex (CD36/β1-integrin/CD47) on the cell membranes [11], [33]. However, opinions still differ about the involvement of 24-OH in amyloid precursor protein (APP) processing and, consequently, in Aβ production. It has been shown that, in neuronal SK-N-BE cells, 1 µM 24-OH enhances the expression and activity of β-secretase, the main enzyme involved in the APP amyloidogenic pathway, and consequently leads to increased Aβ generation and accumulation [12]. Conversely, it has been reported that 24-OH (5–10 µM) can inhibit Aβ plaque formation by favoring the non-amyloidogenic APP pathway [34], [35]. Another study suggests that 24-OH (1–10 µM) is able to reduce Aβ production by downregulating the intracellular APP trafficking [14]. Of note, 24-OH is a selective positive allosteric modulator of N-methyl-D-aspartate receptors (NMDARs) that are critical to the regulation of excitatory synaptic function. 24-OH (2 µM) potentiates NMDAR-mediated responses and restore cognitive deficit in rodents treated with NMDAR channel blockers [36].

The role of 7-K in modulating senile plaque formation has not yet been investigated in depth. It is reported to enhance Aβ insertion into the lipid bilayer, by decreasing intramolecular cohesive interactions [37]. Moreover, it has been demonstrated that 7-K (125 uM) induces mitochondrial dysfunction in the neuronal PC12 cell line, leading to cell death, and also that incorporation of 7-K into lipid rafts domains of plasma membranes triggers apoptotic signaling [38].

Interestingly, the correlation of tau pathology with 24-OH levels, specifically with modulation of the enzyme CYP46A1, has been investigated by injecting the adeno-associated virus-CYP46A1 vector into the hippocampus of THY-Tau22 mice; these animals are considered to be a model of AD-like tau pathology. It was found that, in the hippocampus of these mice, levels of both 24-OH and CYP46A1 are lower than in control mice. After injection of the vector, CYP46A1 and 24-OH content were increased to control levels, and cognitive deficits were remedied, whereas tau hyperphosphorylation and associated gliosis were unaffected, indicating that CYP46A1 may be a relevant therapeutic target for tauopathies, and especially for AD [17]. Of note, very recently, to test the efficacy of reducing tau levels in the brain, antisense oligonucleotides that selectively decrease tau mRNA and protein have been identified in mice carrying human tau. Lowering total tau expression was not only capable of preventing and reversing tau pathology, but survival was significantly extended and neuronal loss abrogated, despite the fact that tau is an abundant and likely important protein in the brain [39].

For these reasons it appears important to emphasize that the close correlation among NFT formation, synaptic and neuronal failure, and cognitive decline may underline the importance of identifying new therapies targeting tau.

Recently, the connection between AD and sirtuins has received much attention, and in particular it is now known that the deacetylase SIRT1 takes part in neuroprotective pathways in AD, by exerting different beneficial effects: i) it is closely linked to inflammation, indeed it regulates the anti-inflammatory response, by inhibiting the transcription factor NF-κB through deacetylation of the Lys^310^ residue of RelA/p65, and by activating the nuclear liver X receptor (LXR) via deacetylation of Lys^432^; ii) it activates the transcriptional co-activator peroxisome-proliferator-activated receptor γ co-activator 1α (PGC1α), which plays a key role in the regulation of cellular energy metabolism and in the antioxidant response; iii) its activation reduces Aβ plaques, thanks to its ability to overexpress the α-secretase gene *ADAM10*; iiii) it prevents NFT formation by deacetylating tau and inducing its degradation [18], [40].

Importantly, it has now been established that, in AD-affected brains, SIRT1 levels are markedly reduced, in parallel with the accumulation of hyperphosphorylated tau [7], [41]. In particular, previous results obtained in the authors’ laboratory, on autopsy specimens from the cortex of AD brains, classified by the Braak staging system of neurofibrillary pathology, have demonstrated that the expression levels of SIRT1 are markedly reduced with the progression of AD, i.e. with the accumulation of hyperphosphorylated tau [7].

Taking into account that, during AD progression, 24-OH and SIRT1 levels decrease on the one hand, and on the other hand NFT accumulates, it appeared of interest to determine whether the loss of 24-OH and SIRT1 could be responsible for the hyperphosphorylation of tau.

The aim of this study was to investigate in SK-N-BE cells the role in tau pathology of two oxysterols present in AD brain, 24-OH and 7-K, in particular concentrating on their ability to modulate tau phosphorylation, in some way affecting the formation of NFT. This appears to be an aspect that no studies have yet considered, since reported data relate almost exclusively to the role of oxysterols in amyloid pathology. Moreover, it was decided to study the potential ability of 24-OH and 7-K to stimulate both expression and synthesis of SIRT1.

In this experimental model, 1 µM 24-OH has been proved to exert a protective effect on SK-N-BE cells, by upregulating both expression and synthesis of SIRT1 (Fig. 1), consequently preventing the accumulation of hyperphosphorylated tau, suggesting that 24-OH could be protective against the aberrant formation of insoluble tau aggregates in neurons. The reduced levels of phospho-tau following treatment with 24-OH seems to be due to SIRT1-dependent deacetylation of tau (Fig. 3A). Indeed, total tau levels were also significantly reduced after cell treatment with 24-OH (Fig. 3). It is hypothesized that 24-OH could contribute to tau degradation by favoring SIRT1-dependent deacetylation of tau; tau would thus become more susceptible to ubiquitination and subsequent proteasomal degradation, leading to total tau reduction in neurons [42]. Taking into account that the dysfunction of protein degradation mechanisms has been proposed to play an important role in AD [43], [44], these results could represent the starting point for a new tau-lowering therapy. Another explanation for the prevention of tau hyperphosphorylation by 24-OH could be the potential ability of this oxysterol to inhibit the activity of the kinases responsible for tau phosphorylation, i.e. glycogen synthase kinase 3β (GSK3β), cyclin-dependent kinase 5 (CDK5), protein kinase A (PKA), and microtubule affinity regulating kinase (MARK) [45]. Furthermore, in addition to the reduced phosphorylation of tau, the increased tau dephosphorylation could also be responsible for the effect induced by 24-OH [46], together with other post-translational modifications of tau, such as tau N-glycosylation [47], or tau processing through proteolysis by caspases, calpains, thrombin, and cathepsin [44]. Unlike 24-OH, 7-K did not modulate SIRT1 levels in SK-N-BE cells (Fig. 2) and, as expected, it did not affect tau levels (Fig. 4).

The subsequent experiments were planned to verify whether a redox imbalance might modulate the SIRT1-dependent axis favoring cell survival. Indeed, although severe oxidative stress can be deleterious to cells, moderate levels of ROS may function as signals to promote cell proliferation and survival [48]. In the present study it was observed that 24-OH, unlike 7-K, causes an early increase of ROS steady-state levels in SK-N-BE cells (Fig. 5A). To confirm the involvement of ROS production in the 24-OH-dependent activation of SIRT1, and in the consequent inhibition of hyperphosphorylated tau accumulation in neurons, cells were incubated with apocynin or with rotenone. As expected, by blocking the intracellular generation of ROS, the protein levels of SIRT1 were no longer upregulated (Fig. 5B) and, consequently, tau phosphorylation levels rose (Fig. 5C). Further confirmation of the involvement of SIRT1 in the 24-OH-dependent neuroprotective effects was obtained by employing the gene silencing technique, through which it was demonstrated that SIRT1 upregulation by 24-OH is necessary to prevent tau hyperphosphorylation (Fig. 5D).

Of note, tau phosphorylation is typically associated with a toxic phenotype, as NFT are extensively hyperphosphorylated in human AD brains and they are associated with neuronal death [49].

The neuroprotective action of 24-OH is strongly supported by in vivo evidence obtained following the ICV injection of 24-OH or 7-K in hTau mice that develop tau pathology only after Aβ monomer administration: 24-OH, unlike 7-K, induces SIRT1 protein levels and prevents the phosphorylation of tau (Fig. 6).

The results reported here show, for the first time, that the neuroprotective effect of 24-OH, in contrast to 7-K, is due to its ability to increase levels and activity of SIRT1, which prevents tau phosphorylation (Graphical abstract), although other molecular mechanisms potentially involved in this effect should be investigated.

## 5 Conclusion

Based on these results, it would clearly be of clinical importance to prevent the loss of 24-OH in the brain by means of its administration, in order to stimulate expression of SIRT1 in the long term with the consequent prevention of NFT accumulation and tau pathology. These findings thus offer new avenues for the development of innovative prevention strategies for AD.

## Funding

The work was supported by the Compagnia di San Paolo (CSTO167048) and the University of Turin (RILO2016).

## Conflict of interests

The authors declare that they have not conflict of interests.
